# Successful Commando procedure using the superior transseptal approach in a high-risk case

**DOI:** 10.1186/s44215-025-00220-7

**Published:** 2025-09-02

**Authors:** Hiroki Moriuchi, Mamoru Orii, Takayuki Fujii, Kohei Narayama, Nobuhiro Shimabukuro, Akihiko Yamauchi

**Affiliations:** Department of Cardiovascular Surgery, Yuai Medical Center, Yone 50-5, Tomigusuku, Okinawa Japan

**Keywords:** Commando procedure, Superior transseptal approach, Annular enlargement

## Abstract

**Background:**

The Commando procedure, which involves replacement of both the aortic and mitral valves along with reconstruction of the intervalvular fibrous body, is technically demanding. Commando procedure typically performed via an incision extending from the ascending aorta to the roof of the left atrium. However, in patients with extensive adhesions due to prior cardiac surgery, adding a superior transseptal incision can provide good exposure and reduce the risk of surrounding tissue injury.

**Case presentation:**

A 48-year-old woman with end-stage kidney disease on dialysis, diabetes mellitus, bilateral leg amputations from critical limb ischemia, and chronic steroid use presented in cardiogenic and septic shock. The patient had undergone mitral valve repair and coronary bypass surgery using saphenous vein grafts. Echocardiography revealed severe aortic and mitral valves stenosis. Given the extensive adhesions and complex anatomy, the Commando procedure was performed using a superior transseptal approach. A 25-mm MITRIS was implanted in the mitral position, and a 25-mm INSPIRIS in the aortic position. A tailored oval-shaped patch made of bovine pericardium was used to reconstruct the intervalvular fibrous body. The patient recovered without major complications.

**Conclusion:**

The superior transseptal approach provided excellent exposure and a stable operative field, facilitating standardized surgical maneuvers throughout the Commando procedure.

## Introduction

In the Commando procedure, aortotomy and left atrial roof incision are commonly used approach. However, the addition of the superior transseptal approach is particularly effective in cases with severe adhesions. Although the Commando procedure is technically challenging, we report a successful case performed using the superior transseptal approach.

## Case presentation

**Fig. 1:**
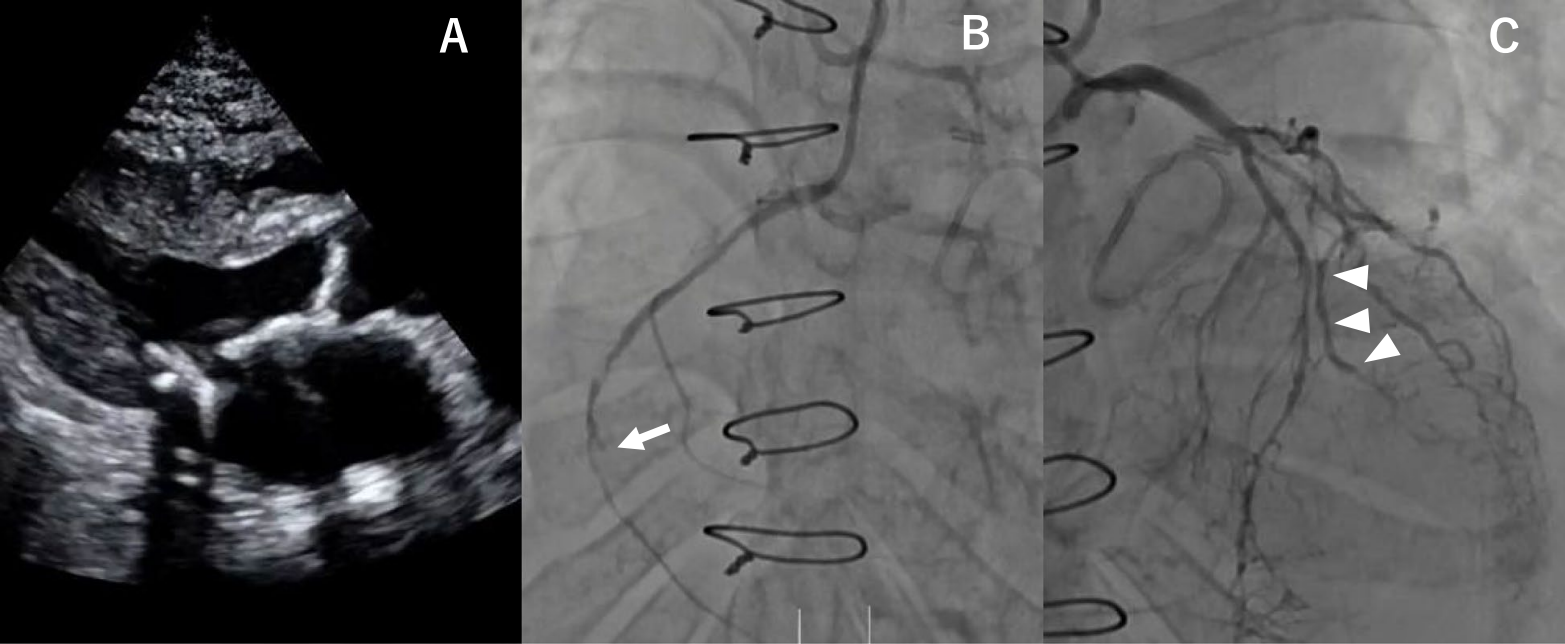
**A** Echocardiography revealed calcification with restricted mobility of both the aortic and mitral valves. **B** Coronary angiography showed occlusion of the right coronary artery (white arrow) and **C** the previous great saphenous vein graft(white arrow head)

A 48-year-old woman with end-stage renal disease on hemodialysis, diabetes mellitus, bilateral leg amputations due to critical limb ischemia, and chronic steroid use was transferred to our hospital in cardiogenic and septic shock. Septic shock was caused by a lower extremity ulcer, from which Methicillin-Resistant Staphylococcus aureus was cultured. The patient’s body measurements were height 150 cm, body weight 50.0 kg, and BSA 1.43 m^2^. Laboratory tests showed WBC 12,000/µL, hemoglobin 9.7 g/dL, platelets 71,000/µL, and CRP 5.5 mg/dL. The patient’s surgical history included mitral valve plasty using a 26-mm Memo 4D annuloplasty ring (LivaNova, London, UK), and coronary artery bypass grafting (CABG), consisting of an aorto–saphenous vein graft (SVG) to the high lateral branch and posterior descending artery, as well as a posterior lateral artery with Y-composite graft, performed at another institution. At the time of admission, the patient was intubated and supported with extracorporeal membrane oxygenation (ECMO) and an intra-aortic balloon pump (IABP). Coronary angiography showed three-vessel disease and an occluded previous bypass graft (Fig. 1A), but hemodynamics remained stable with ECMO and IABP. Echocardiography under ECMO support and inotropic agents (dopamine 3 µg/kg/min, dobutamine 5 µg/kg/min, noradrenaline 0.02 µg/kg/min) revealed low-flow, low-gradient severe aortic stenosis (aortic valve area 0.4 cm^2^, peak velocity, 3.5 m/s, mean pressure gradient, 35 mmHg, stroke volume index, 22 mL/m^2^), severe mitral stenosis (mitral valve area, 0.5 cm^2^, mean pressure gradient, 11 mmHg), and severe tricuspid regurgitation (tricuspid regurgitant pressure gradient 37 mmHg). The left ventricular end-diastolic diameter was 35 mm, and the left ventricular ejection fraction was 50% (Fig. 1B). After thorough discussion with the family, and considering their strong preference, we proceeded with surgical intervention. Commando procedure was planned preoperatively because severe adhesions and the need for annular enlargement were anticipated before surgery.

**Fig. 2:**
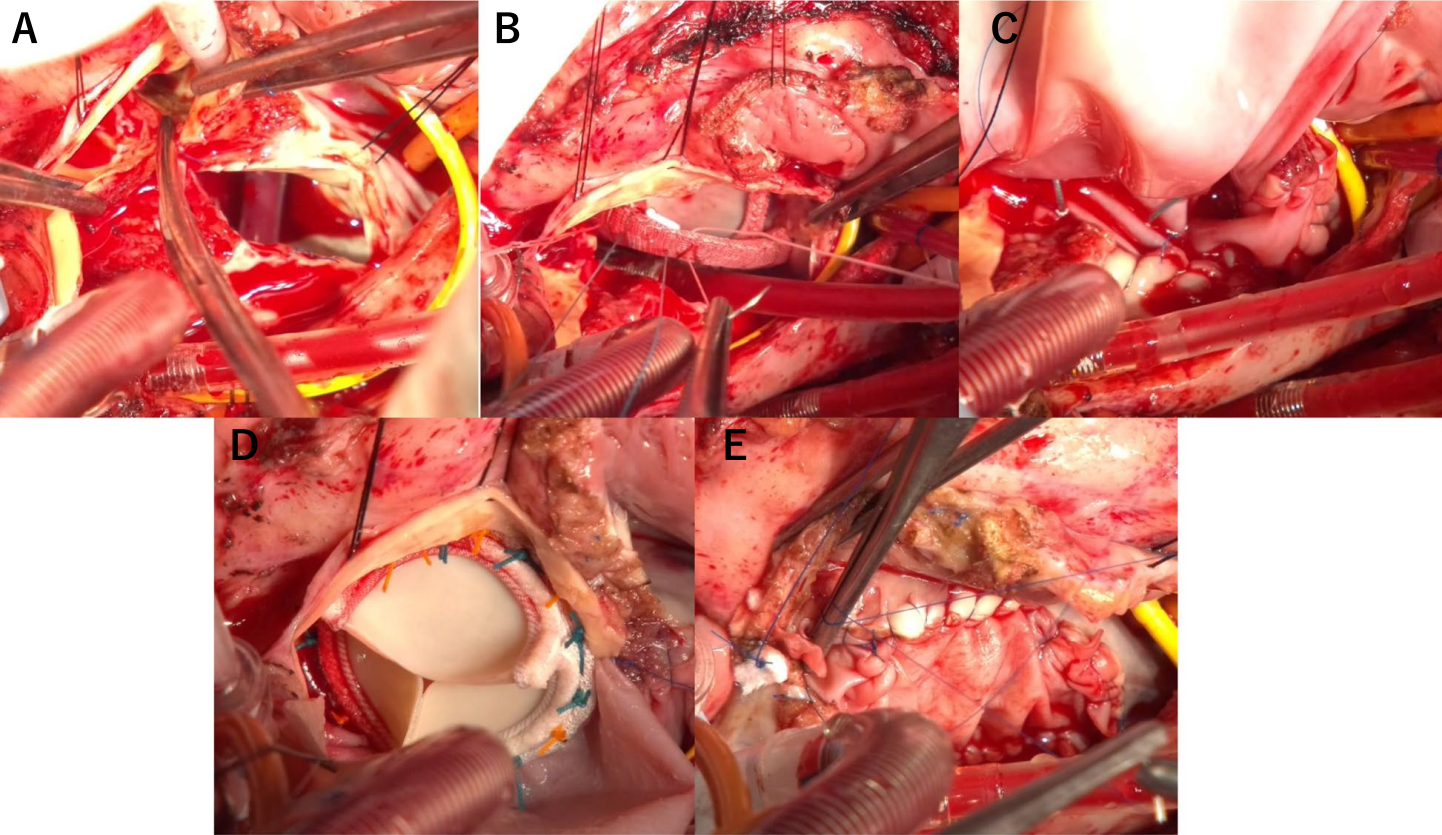
Intraoperative photos. **A** The aortic incision was connected to the atrial septum and left atrial roof incision. **B** The MITRIS valve was sutured posteriorly to the native annulus and anteriorly to the patch. **C** Patch closure from left atrial roof to atrial septum. **D** The INSPIRIS valve was sutured using the patch. **E** Additional patch was used to close the right atrium

Under general anesthesia, a median resternotomy was performed, and the left internal thoracic artery (LITA) was harvested in a skeletonized fashion using a harmonic scalpel (Ethicon Endo-Surgery, Inc., Cincinnati, OH, USA). LITA was anastomosed to the previously used SVG with 7–0 polypropylene suture under ECMO support. After cardiopulmonary bypass was established via ascending aortic arterial perfusion and bicaval venous drainage, the ECMO cannulas were removed. A left ventricular vent was placed via the right upper pulmonary vein. Under full cardiopulmonary bypass, a right atriotomy was performed, and a retrograde cardioplegia catheter was inserted into the coronary sinus. Following aortic cross-clamping, retrograde cardioplegia was administered, resulting in cardiac arrest. An oblique aortic incision and a superior transseptal incision was made to expose the aortic and mitral valves. After decalcification of the aortic valve, a 21-mm INSPIRIS sizer could not be passed through the annulus, therefore, we decided to proceed with the Commando procedure. The aortotomy was extended between the left and noncoronary commissures, across the aortic annulus and into the mitral annulus after removal of the Memo 4D ring, where it was connected to the superior transseptal incision (Fig. 2A). A 25-mm MITRIS (Edwards Lifesciences, Irvine, CA, USA) was implanted in the native mitral annulus using pledgetted interrupted sutures, with the suture line covering approximately two-thirds of the prosthetic circumference (Fig. 2B). An oval-shaped bovine pericardial patch was used to complete the MITRIS valve implantation and reconstruct the IVF. After sizing the aortic annulus, a 25-mm INSPIRIS (Edwards Lifesciences, Irvine, CA, USA) was implanted using the pledgetted sutures (Fig. 2C, D). The upper half of the patch was used for the ascending aorta, and the lower half for the roof of the left atrium and atrial septum. Tricuspid annuloplasty was performed using an MC3 ring (Edwards Lifesciences, Irvine, CA, USA), and an additional patch was used to close the right atrium (Fig. 2E).

**Fig. 3:**
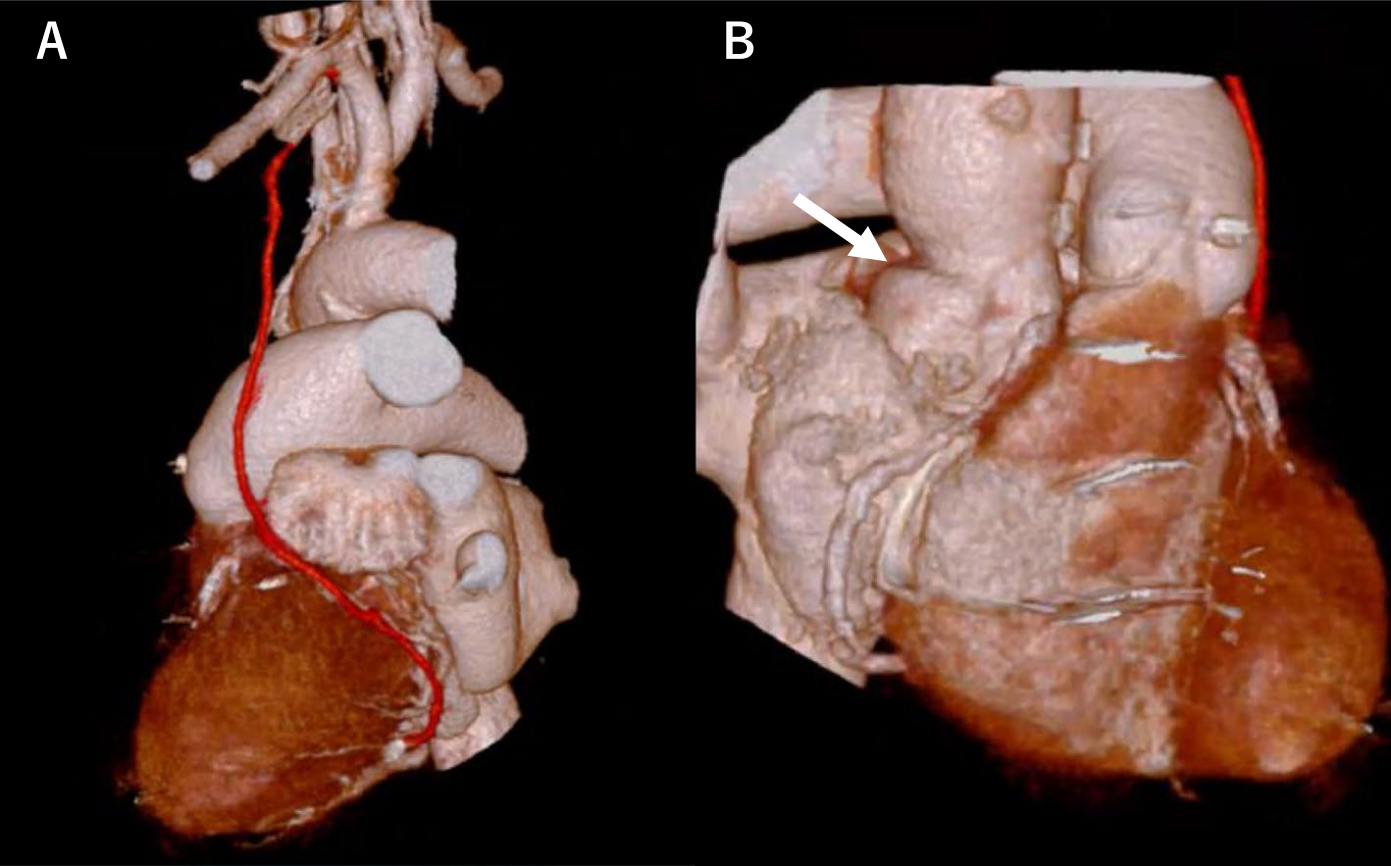
Postoperative computed tomography revealed (**A**) graft patency and (**B**) patch enlargement (white arrow)

The patient was successfully weaned from ECMO on postoperative day (POD) 0 and from IABP on POD 1. She was extubated on POD 5. Postoperative echocardiography showed well-functioning prosthetic valves with no paravalvular leakage, CT confirmed good patency of the bypass graft and proper morphology of the reconstructed area (Fig. 3). The patient was discharged to a rehabilitation facility on POD 14.

## Discussion and conclusion

The Commando procedure involves aortic valve replacement, mitral valve replacement and reconstruction of the intervalvular fibrous body. Indications for the Commando procedure include extensive calcification of the fibrous skeleton, abscess involving the aortic root or mitral annulus, prior aortic and mitral valve replacements, and patient–prosthesis mismatch [1]. The standard surgical approach in the Commando procedure commonly involves a continuous incision from the ascending aorta to the roof of the left atrium. However, a combined superior transseptal approach is particularly effective in cases with dense adhesions due to prior cardiac surgery. A wide incision extending from the atrial septum to the left atrial roof and the aortic root is essential and this approach offers excellent exposure of the mitral valve and intervalvular fibrous body, while minimizing the need for extensive dissection and reducing the risk of injury to surrounding structures [2].

The size of the mitral valve prosthesis is selected to ensure that the anterior portion of the sewing cuff lies below the plane of the aortic annulus. To prevent left ventricular outflow tract obstruction (LVOTO), it is important to avoid oversizing the mitral valve prosthesis. In addition, less than one-third of the mitral annulus was sutured to the patch to minimize the risk of LVOTO. The width of the patch is tailored to be approximately 1 cm greater than the linear distance between the left and right fibrous trigones [3]. Due to the risk of hemorrhage, a mechanical valve was not chosen, and a sutureless valve was avoided to ensure durability and procedural reliability.

David reported that the operative mortality of the Commando procedure was 13.2%, and the survival rates at 1, 10, and 20 years were 81.8%, 51.1%, and 23.7%, respectively [1]. The high operative mortality reflects the fact that patients undergoing the Commando procedure are often in critical condition due to severe infective endocarditis or complex reoperative cardiac surgery.

In this case, due to severe adhesions, the superior transseptal approach was chosen to ensure adequate surgical exposure. This approach provides a good operative field, which helps standardize the surgical maneuvers of the Commando procedure. In our case, no major perioperative complications occurred, however, long-term follow-up is necessary.

## Data Availability

The data underlying this article will be shared on reasonable request to the corresponding author.

## Abbreviations

CABG: Coronary artery bypass grafting
ECMO: Extracorporeal membrane oxygenation
IABP: Intra-aortic balloon pump
CT: Computed tomography
LITA: Left internal thoracic artery
SVG: Saphenous vein graft
POD: Postoperative day
LVOTO: Left ventricular outflow tract obstruction
